# Sequential mountings of a possible immature male by an adult male in Victoria’s Riflebird (*Ptiloris victoriae*)

**DOI:** 10.52732/TRDN8657

**Published:** 2023-12-11

**Authors:** Thomas MacGillavry, Leonida Fusani

**Affiliations:** 1 Konrad Lorenz Institute of Ethology, University of Veterinary Medicine, Vienna, Austria; 2 Department of Behavioural and Cognitive Biology, University of Vienna, Vienna, Austria

**Keywords:** courtship, display, sexual behaviour, mounting, copulation, birds of paradise

## Abstract

Few studies have offered detailed descriptions of copulatory behaviours in the birds of paradise (family Paradisaeidae) and systematic investigations of their sexual behaviours are rare. We recorded courtship behaviours of Victoria’s Riflebird *Ptiloris victoriae* in the Atherton Tablelands in Queensland, Australia using motion triggered cameras and report a rare case of three sequential mountings by an adult-plumaged male. While the recipient of these mountings performed female-typical sexual behaviours, it also briefly performed a male courtship display behaviour, suggesting that it may be an immature male. This observation raises several questions about the courtship behaviour of this species. Females may, for instance, occasionally solicit multiple copulations from preferred males to maximize the amount of transferred sperm. Another intriguing possibility is that immature males tolerate being mounted and potentially even mimic female sexual behaviours when learning from the displays of adult males. We also describe a novel adult male sexual behaviour, namely ‘nape-pecking’.

## Behaviours / Natural History Observations

Victoria’s Riflebird *Ptiloris victoriae* is a polygynous species of bird of paradise (family Paradisaeidae) endemic to the Wet Tropics of Far North Queensland in Australia. Adult males defend dispersed display perches during the breeding season (roughly between August and November), on which they perform elaborate courtship displays involving a static *circular-wings* posture and a dynamic *alternating wing-clap* display [1]. As is typical of most birds of paradise and other species exhibiting polygynous, non-resource-based mating systems, males acquire their adult, nuptial plumage following an extended immature period, during which they are drably coloured and effectively indistinguishable from females. In Victoria’s riflebird, this period is thought to last for approximately seven years [2], after which males begin to develop their characteristic black and structurally coloured feathers. While the general features of the display behaviour of this species are well known, its copulatory behaviour has rarely been documented.

In our ongoing study of Victoria’s Riflebird, every recorded mounting occurred as a single event, after which the receiver immediately left the display perch (TM unpublished data). Here, we report a rare instance where a female-plumaged receiver remained on the display perch for three consecutive mountings by a courting adult male. The receiver exhibited typical female sexual arousal behaviours such as wing-fluttering [3] and crouching [4]. Surprisingly, the same bird briefly performed a courtship display behaviour typical of males, suggesting that the receiver may have been an immature male.

The following behaviours were recorded at 3:37pm on August 26, 2023 using a motion-triggered camera (Browning Recon Force Advantage HD, 2018) installed near a display perch at lake Barrine in the Crater Lakes National Park in the Atherton Tablelands (Queensland, Australia; permit number: P-PTUKI-100257238).

At 0s, the resident adult male performs the *circular-wings* display directed at a receiver not visible in the frame. At 16s, a female-plumaged individual lands on the display perch, after which the adult male immediately performs the *alternating wing-clap* display [1]. The female-plumaged bird closely observes the displaying adult male as is typical in this species, and begins wing-fluttering and crouching at 23s—typical indicators of sexual interest in birds [3][4]. The displaying adult increases the rate of wing-claps and mounts the female-plumaged bird at 37s for approximately 3s. Upon dismounting, the adult male continues wing-clapping at a high rate (approximately three claps per second) and the female-plumaged bird remains attentive on the display perch.

At 46s, the female-plumaged bird briefly fully extends its left wing and partially extends its right wing—typical of the *alternating wing-clap* display—and lowers it as the adult male continues displaying. At 53s, the adult male mounts again for approximately 5s, appears to peck the head of the mounted bird three times, then again continues performing the alternating wing-clap display. View of the female-plumaged bird prior to this mounting was obstructed by the adult male, and so it was not possible to determine if the female-plumaged bird was engaging in any solicitation behaviour.

The female-plumaged bird is seen wing-fluttering again at 67s, and crouches at 70s, after which the adult male mounts for a third time. This third instance of mounting lasted for 7s, during which the adult male vigorously pecked at the head of the mounted female-plumaged bird seven times. Several of these pecks were hard enough to produce a sound audible in the recorded video footage.

At 78s, the adult male dismounts and continues to perform the *alternating wing-clap* display until the female-plumaged bird leaves the display perch at 82s. The adult male then continues to perform the *circular-wings* display for 37s facing the direction the female-plumaged bird flew until the end of the video at 120s.

Since, in each observed mounting, the adult male leaned backwards and pushed the tail of the recipient aside in an attempt to achieve cloacal contact, they can be considered as attempted copulations, though we have no evidence of successful sperm transfer.

**Figure 1:**
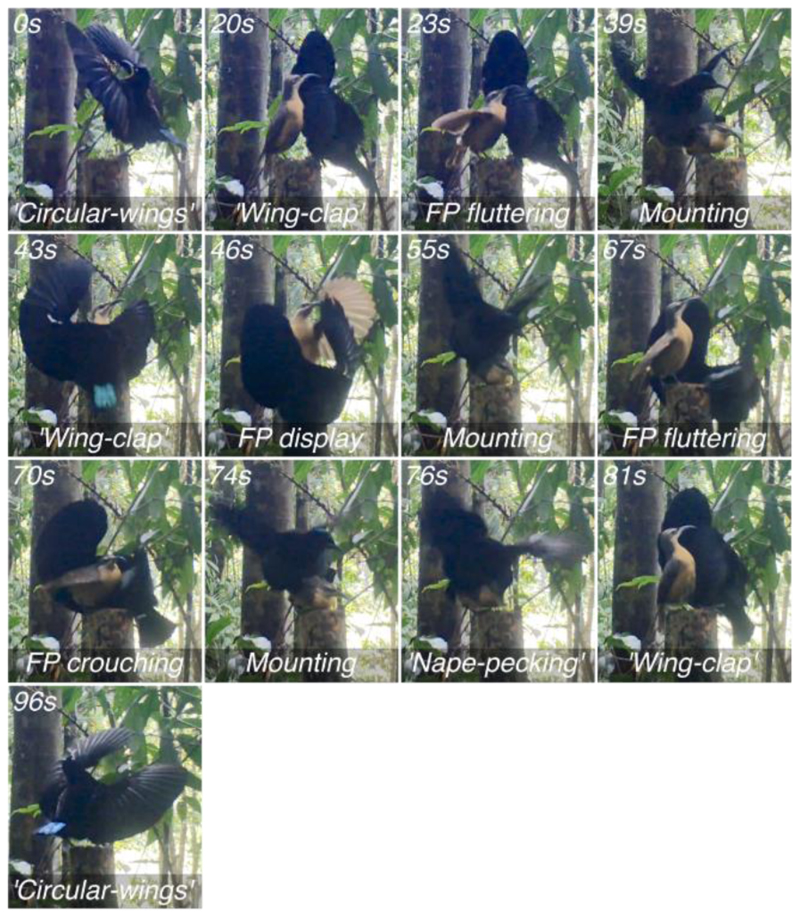
The annotated sequence of behaviours described in the present article as follows: the circular-wings display (20s), alternating wing-clap display (20s), wing-fluttering (23s), mounting (39s), alternating wing-clap display (43s), display behaviour by female-plumaged bird (46s), mounting (55s), alternating wing-clap display (67s), crouching by female-plumaged bird (70s), mounting (74s), vigorous nape-pecking by the adult male (76s), alternating wing-clap display (81s), and circular-wings display (96s). The time stamps indicate the time at which the displayed frame occurred in the video in order to visualize the behaviours as clearly as possible. Exact times at which each behaviour occurred in the video are shown in the main text. Annotations beginning with ‘FP’ refer to behaviours produced by the female-plumaged bird.

Still frames of the video footage discussed above and timestamps at which they were taken are shown in figure 1. The full video is available here: https://youtu.be/SpfbK9tgzbo.

## Functional Hypotheses

The observations presented here raise several intriguing questions about the sexual behaviour of Victoria’s Riflebird. Since the female-plumaged bird was un-banded and could not confidently be sexed, the following discussion will consider two alternative scenarios depending on the assumed sex.

First, if the mounted bird was female, one adaptive explanation for multiple copulation is that it allows females to maximize the transfer of sperm from preferred males, thus reducing the need to re-visit display perches. However, since this was the only instance of multiple mountings recorded throughout a five-month study period, it is unlikely to be an adaptive strategy for females—or at least not one that is commonly employed.

An alternative hypothesis entails that immature males visit the display perches and attend the courtship displays of adult males as they benefit through production learning of the motor patterns used in display [5], much like how song is learned among vocal learning birds. Unlike socially monogamous songbirds, however, where vocal signatures are often learned from a parent tutor [6][7], polygynous species lacking male parental care may learn visual motor patterns by seeking out adult male tutors during the immature period.

Associations between immature or subordinate males and courting adults have been studied in other polygynous bird species, such as Spotted Bowerbirds *Chlamydera maculata* [8], though subordinate males in such systems have also been observed attempting to ‘steal’ copulations from adults [9], suggesting that the benefits of such associations extend beyond learning. In birds of paradise, similar associations between adult and immature males may occur in the Magnificent *Cicinnurus magnificus* and Wilson’s Bird of Paradise *C. respublica* (Edwin Scholes, personal communication), though this has not yet been investigated in detail. It may, however, be a widespread feature of polygynous birds with delayed plumage maturation in males, that immature males invest significantly in display practice—as is also the case in Victoria’s Riflebird [1]—including visitations to the display areas of adults.

Furthermore, if the female-plumaged individual described here was indeed male, then the tantalizing possibility emerges that immature males perform female solicitation displays, such as wing-fluttering and crouching in order to presumably avoid an aggressive response from the courting adult male. One piece of evidence to support this hypothesis is that adult males occasionally chase female-plumaged birds away from the display perch when they refuse to attend the display (*e.g*., by, instead, sitting in the canopy close to the display perch; TM, personal observation). Both immature males as well as females may thus need to indicate some degree of interest in order for adult males to tolerate them at the display perch. This explanation, however, contrasts with the fact that the adult male in the present footage did not chase the female-plumaged bird away when it performed a male-typical display behaviour. Furthermore, female-like wing-fluttering has been described in courting adult males of other bird of paradise species [10], though its function is unclear. Overall, the behaviours exhibited by the receiver in the present footage are insufficient for confident sexing.

While the sex of the female-plumaged bird could not be determined, future research may yet allow for sexing, as males are able to hyper-extend the manus substantially when the wings are raised during display, and it is not yet known whether females are also capable of doing so (TM unpublished data). However, since accounts of male-typical courtship displays of female birds of paradise in the wild are exceedingly rare [2], it is likely that our observation involves two males. However, it is worth noting that unsexed, female-plumaged Magnificent Riflebirds *P. magnificus* typically partially raise (but do not hyper-extend) the wings while attending adult male courtship displays (see Macauley Library accession number: ML481867).

While the aforementioned hypotheses hone in on the behaviour of receivers, our observations also highlight previously undescribed behaviours of adult male Victoria’s Riflebirds. Previous descriptions as well as extensive video recordings of courtship displays in this species show that adult males continue to display following mountings and possibly also copulations [1] (TM unpublished data). The observations presented here thus suggest that adult males will attempt multiple mountings if receivers were to continue soliciting—a behaviour that likely functions to enhance sperm competition.

Finally, we also report a previously undescribed male peri-copulatory behaviour [11] in this species, namely ‘*nape-pecking*’. While variations of this behaviour have previously been described in other birds of paradise, including, for example, Greater Bird of Paradise *Paradisaea apoda*, Magnificent Bird of Paradise, Twelve-wired Bird of Paradise *Seleucidis melanoleuca*, and Wallace’s Standardwing *Semioptera wallacii* [2], it had never been documented in Victoria’s Riflebird. However, whether this behaviour functions as a courtship display or as a form of sexual coercion remains unknown.

Since long-term banding projects of wild Victoria’s Riflebirds are logistically challenging, particularly due to the difficulties of trapping and observing a sufficient sample of female-plumaged individuals in which sex is known, future studies of habituated populations may be fruitful at testing the hypotheses presented here.
